# The physical therapy profile questionnaire (PTPQ): development, validation and pilot testing

**DOI:** 10.1186/1756-0500-4-362

**Published:** 2011-09-19

**Authors:** Janine Margarita R Dizon, Karen Grimmer-Somers, Saravana Kumar

**Affiliations:** 1 International Centre for Allied Health Evidence, University of South Australia City East Campus, North Terrace Adelaide, SA 5000 Australia; 2 University of Santo Tomas Espaňa Ave, Manila 1008 Philippines

## Abstract

**Background:**

Country by country similarities and differences in physical therapy practice exists. Therefore, before updates in practice can be provided, such as trainings in evidence-based practice, it is necessary to identify the profile and nature of practice in a given country or setting. Following a search of the international literature, no appropriate tool was identified to collect and establish data to create the profile of physical therapy practice in the Philippines. We therefore developed, validated and pilot tested a survey instrument which would comprehensively describe the practice of physical therapy in the Philippines

**Findings:**

We used a mixed methods design to answer our study aims. A focus group interview was conducted among a group of physical therapists to establish the content and contexts of items to be included in the survey instrument. Findings were amalgamated with the information from the literature on developing survey instruments/questionnaires. A survey instrument was drafted and named as the Physical Therapy Profile Questionnaire (PTPQ). The PTPQ was then validated and pilot tested to a different group of physical therapists.

The final version consisted of five separate parts namely (A) *General information and demographics, (B) Practice Profile, (C) Treatment Preferences, (D) Bases for clinical work and (E) Bases for educational/research work*. At present the PTPQ is relevant to the Philippines and could be used by any country which has a similar nature of practice with the Philippines.

**Conclusion:**

The *Physical Therapy Practice Questionnaire (PTPQ) *was shown to have good face and content validity among the Filipino physical therapists and their context of practice. It has also been found to be useful, easy to administer tool and in a format appealing to respondents. The PTPQ is expected to assist comprehensive data collection to create a profile of physical therapy practice in the Philippines.

## Background

Physiotherapy or Physical therapy (used interchangeably) is a key allied health profession practiced around the world. It is defined by the World Confederation for Physical Therapy as a profession that *"provides services to individuals and populations to develop, maintain and restore maximum movement and functional ability throughout the lifespan. This includes providing services in circumstances where movement and function are threatened by ageing, injury, disease or environmental factors. Functional movement is central to what it means to be healthy" *[1].

There are country-by-country differences in the way physical therapy is practiced, both in terms of what treatments are provided, and how patients gain access to physical therapy services. These differences reflect specific country legislative requirements and constraints, physical therapy training, historical practices, culture and local patient needs [2]. In a few countries (Canada and Australia for instance), physical therapists have been legislated as first contact practitioners (meaning they do not need a referral from a doctor to provide treatment). However in the Asian region, physical therapists usually treat patients following a referral from medical doctors. The Philippines is one country where physical therapists require a referral from a doctor before they can treat patients. This referral usually contains a prescription for treatment which the physical therapist may be bound to follow.

These variations in practice place physical therapists in different legal and professional status, depending on whom they work with or where they work [2-5].

Despite whether they are first contact practitioners or not, physical therapists are faced with challenges underpinned by changes in the way that health services are delivered and funded. The World Health Organization reports key factors which drive changes in health services internationally, including research evidence, cost constraints, ageing populations, the impact of technological advances (e.g., pharmaceuticals), increased consumer expectations and knowledge, stakeholders' needs for improved health outcomes, and changes in health care tasks [2,6]. To respond to these changes, physical therapists require regular continuing professional education, training and up skilling [2,5].

In the Philippines, one of the many challenges that physical therapists face is the recent change in the focus of disease internationally. The focus has significantly shifted based on the needs of the society; from the management of the sequelae of acute and chronic conditions, to prevention of lifestyle-driven chronic diseases [7]. Chronic conditions such as cardiovascular diseases, chronic obstructive pulmonary diseases and diabetes currently account for almost half of the deaths in the Philippines [8]. Ninety percent of Filipinos have one or more of the risk factors related to the development of chronic disease such as physical inactivity, smoking, hypertension and obesity [8]. Prevention of mortality and disease risk factor modification are areas where physical therapists can play a significant role, in promoting physical activity and healthier lifestyles. However, physical therapists still play a major role in managing the sequelae of established chronic diseases. For instance, the prevalence of disability and chronic musculoskeletal diseases such as arthritis, among older adults in the Philippines has increased through the years [9] and the management of these conditions requires the application of best-practice physical therapy services focusing on functional re-training to optimize independent mobility.

There is no published information on the current practice of physical therapy in the Philippines. Thus nothing is documented about how Filipino physical therapists have responded to the challenges of applying evidence-based practices to the prevention and management of chronic disease and its sequelae, and how they deal with continual changes to the evidence-based underpinning best practice treatments. Thus to ensure that Filipino physical therapists are provided with the requisite supports to assist them to provide best practices, it is important to establish the current profile of physical therapy practice in the Philippines, so that appropriate educational activities and supports can be provided to improve the quality of Filipino physical therapy services.

Following a search of the international literature using keywords to search databases, we identified no appropriate survey instrument with which to establish a profile of physical therapy practice in the Philippines. We therefore developed, validated and pilot tested a survey instrument which we believed would comprehensively describe the current profile of practice and the basis for treatment decisions by physical therapists in the Philippines. This paper describes the developmental processes for this survey tool.

## Methods

### Research Design

A mixed methods research design was undertaken. Mixed methods research draws on the strengths of both qualitative and quantitative research designs [10]. Two approaches were taken: A qualitative approach to develop the contents of the survey instrument, and quantitative research to validate and pilot test the draft survey instrument.

### Ethics

This study was approved by the Research Ethics Committee of the Division of Health Sciences, University of South Australia.

### Procedures

#### Literature search

We searched the literature for a valid instrument that could capture the nature of, and establish the profile of, physical therapy practice in the Philippines (or any other country which can reflect a similar nature of practice). We used the keywords "physical therapy" OR "physiotherapy" AND "profile" to search the following databases: MEDLINE, CINAHL, EMBASE, PEDro and GOOGLE SCHOLAR. However, we found none that was appropriate to answer our objectives. What the literature provided were studies presenting profiles of physical therapy practice in developed countries such as Australia [11-13], the United States [14] and the United Kingdom [15]. We also found one instrument from Thailand specifically focusing on physical therapy treatment of low back pain [16]. There was no other existing instrument that can capture the practice of physical therapy in the Philippines, most especially with regards to bases for treatment decisions. However, these articles found in the search, plus the literature on designing questionnaires [17-20], informed the development of the purpose-built survey instrument in this study.

#### Qualitative research

We conducted a focus group with seven physical therapists (two males, five females) to establish the content and contexts of items which might be included in the survey instrument. We used a combination of homogeneous purposeful sampling and maximum variation sampling to identify the key informants for the study. Key informants are defined as individuals with rich information on the matter being discussed [21]. Homogeneous purposeful sampling was used to target key informants who are in the same profession. They had to be licensed Filipino physical therapists and practicing for at least two years to have a good sense of practice. Maximum variation sampling was then used to identify physical therapists from different geographical locations, areas and setting of practice. These key informants were identified through the network of the Philippine Physical Therapy Association (PPTA). Letters were sent to the identified key informants to invite them to take part in the study. Information on the project, the objectives, their role as participants, and the schedule and venue for the interview were provided. Written informed consents were signed prior to the actual interview.

The focus group was facilitated by the senior researcher (JRD) while field notes were taken by a research assistant (CR). Open ended questions were asked to explore the informants' perspectives of survey content, to establish the questions which would produce a comprehensive profile of practice and basis of treatment selection. The core questions were: *(1) What are the important background characteristics/information about the local physical therapists, in order to present a profile of practice?, (2) What are the treatment procedures delivered by physical therapists to manage patients?, and (3) what are the common or usual bases for selection of treatment procedures in practice? *The focus group was audio taped and transcribed for analysis. Two independent reviewers coded and analyzed the transcribed data using content analysis approach [21] which identified key and subthemes, and exemplar quotations.

#### Development of the draft survey instrument

We amalgamated the information from the literature search on how to establish profiles of practice, with the transcribed focus group data and field notes from the focus group, to develop the draft Filipino survey instrument. We used two methods of triangulation in the process. These were investigator triangulation and data triangulation [22]. These methods of triangulation ensured validity of findings from all sources of information to design the instrument [23].

The initial draft of the instrument was sent to the members of the focus group to check for congruence with their recall of the focus group discussions. Comments were noted from the participants and the draft was revised and returned to focus group participants for checking.

#### Face and Content Validation

We then invited ten physical therapists who are "experts" in different areas of practice (who have not participated in the focus group) to assess the draft of the survey instrument for face (defined as the items or questions in the instrument appear to be measuring what the instrument is suppose to measure [20]) and content validity (defined as having enough items to cover the domain under investigation [20]), in line with the study objectives. We defined our "experts" as individuals with special skills or knowledge derived from extensive experience with sub domains, based on a proficiency scale by Hoffmann in 1998 [24]. The physical therapists who we considered experts should have acquired not only undergraduate training, but post graduate knowledge and skills training (either as a formal education or participation in certification courses or apprenticeship in specialized training institutions) in their specific areas of practice (sports, paediatric, pulmonary, geriatric, physical therapy research and education etc.) and practicing for at least five years. These experts then took part in our Delphi method, which we used to test for face and content validity. The Delphi method is a means of collecting and combining the comments or opinion of a group of experts who do not meet face to face, using information exchanges [25]. We used email exchanges as our mode of communication with the group of experts. The primary author of this study served as the facilitator of this method.

The main domains that we intended to cover were: (1) demographic information which includes educational background, years of practice, area of practice, practice setting and roles performed in practice, (2) treatment preferences which includes all possible treatment interventions provided to patients and (3) bases for treatment choices which includes experience, trainings and many others. Experts were asked to check the relevance of the questions in line with the domains that we aim to cover in the instrument (content validity), and to comment on the manner by which the questions and responses were drafted (face validity).

Once all responses were collected, the facilitator together with the other authors of this study and an independent researcher, summarised, made amendments as appropriate and returned the revised instrument to the panel of experts for their review and feedback. Three iterative rounds were conducted until consensus among the experts was achieved. Consensus was achieved when there were no more major comments received from the expert panel and each one agreed that the survey instrument is ready for distribution.

It might be worthwhile to mention that we did not test for criterion validity (defined as correlation of a scale with an accepted instrument or measure [20]) as there is no existing accepted instrument in creating a physical therapy profile of practice due to existing variations in practice. We did not test for construct validity (defined as measuring a hypothetical construct [20]) as we are not assessing a specific construct, but rather, we are simply intending to seek direct responses from participants about their current work to create a profile of physical therapists and their practice.

#### Pilot Testing

We pilot tested the survey instrument in two ways: 1) paper questionnaire and 2) an online questionnaire using the survey monkey online tool http://surveymonkey.com. Twenty six physical therapists in different areas of practice took part in the pilot testing. This process allowed respondents to comment on the instructions which may have been hard to follow, questions which may have been difficult to understand and required rephrasing and the level of difficulty encountered in answering the questions. Participants were asked to comment and rate on the instrument's usability, ease of administration, comprehensiveness and format using a 1-5 scale (1-lowest, 5-highest). Time taken to complete the instrument was also noted.

#### Data Analysis

The findings from the pilot process were considered, and wording changes were made to the questionnaire as appropriate.

## Results and Discussion

The survey instrument was named the 'Physical Therapy Profile Questionnaire (PTPQ)'. The first instrument version comprised three parts from the major themes arising from the focus group (See Table 1).

**Table 1:** Contents of the draft survey instrument

Themes regarding content of the survey instrument	
1. General Information and profile of practice	The first part is intended to obtain the demographic data, educational background and characteristics of practice of the participants in terms of years in practice, area of practice (musculoskeletal, neurological, pediatric etc) setting of practice (hospital, clinic, and others) and roles performed (clinician, administrator and others).

2. Treatment Preferences	The second part was intended to obtain treatment techniques/procedures used in practice. An option was provided for general (Manual techniques, electrotherapy, general exercises and others) and specific techniques used (Mulligan's techniques, electrical stimulation, Bobath exercises and others).

3. Basis for Treatment Selection	The third part aimed to determine the basis for the physical therapists' treatment selections (undergraduate education, experience, trainings). From the possible reasons or bases for treatment selection in the focus group discussion, a list was created where options to tick one or more choices as applicable were provided.

This study highlights the importance of using the appropriate instrument to collect the intended data for a research project. An instrument that matches the purpose of the research and data intended to be collected should be the primary consideration in the process of identifying or developing a survey instrument. In the larger context of the authors' study on identifying the profile of physical therapy practice in the Philippines, this study on developing the right instrument took the lead.

### Validation and pilot testing

The face and content validation testing of the PTPQ resulted in revisions, which included additional questions, changes to questions and response categories, and clarification of the intent of questions. Some important modifications done in the draft of the PTPQ as a result of the validation were: (1) dividing the question regarding bases for selection of treatment into "*usual clinical and new or unique cases" *as physical therapists draw on different sources in different situations, (2) asking respondents to rank the bases for treatment selection in order to understand more what physical therapists prioritize and use most often, rather than just checking all that applies and (3) allowing multiple answers in some questions such as practice setting, colleagues at work and many others. This process ensured that the survey can collect high quality generalisable data for the project and should therefore uncover the right information from the respondents [19]. If respondents cannot understand the questions in the survey, they tend not to complete the survey which then results to low response rates [26]. It is recommended that questions should be short; using only twelve words or less.

Table 2 presents the results of the pilot testing. In general, the PTPQ was reported to be useful, easy to administer, comprehensive and has good format. The survey takes about 10-25 minutes to answer, depending on which part applies to the respondents. The feedback on utility was incorporated into the instrument, and minor modifications were made, such as formatting and changes in instruction. For instance, each section of the questionnaire was identified using alphabet letters (e.g. *A-General Information, B-Practice Profile*), instead of numbers (*e.g. 1-General Information, 2- Practice Profile*) to avoid confusion with the numbered items in the questionnaire. This made the instructions more specific and clearer to the respondents (e.g. *If you perform ANY clinical work, please proceed to Sections C (Treatment preferences) and D (Basis for clinical work). If you perform ANY educational/research work, please proceed to Sections E (Basis for educational/research work)*.

**Table 2:** Results of pilot testing

Items rated for pilot testing	Average scores based from a scale of 1(lowest)- 5 (highest)	Comments/Suggestions
1. usability	4.43	• PTPQ was general useful • comment on ensuring page numbers are present

2. ease of administration	4.86	• PTPQ was very easy to administer either through paper copies or online • a query on username and password for the online survey was raised

3. comprehensiveness	4.71	• PTPQ was very comprehensive and covers essential information to describe the profile of practice

4. format	4.43	• PTPQ has good format • suggestions to provide prompts on which page to access next especially for those who need to skip the treatment preferences part • suggestion to change the numbers of the PTPQ headings (1. General information, 2. Practice profile etc) to letters (A. General information, B. Practice profile) so as not to confuse with the numbers in the questions and should provide clearer instruction to which section the respondents should proceed next

Pilot testing the PTPQ on representatives of the population to which it is going to be administered is another essential component of the process of questionnaire development. This process enabled a "trial" run of how the data collection will take place and what the views of respondents are in answering a survey instrument. Data entry and coding are recommended components of pilot testing as this allows troubleshooting of possible problems in data management and analysis [19].

### Final instrument (Additional file 1)

After amalgamating all comments from the developmental steps, the final PTPQ was produced. To further ensure that the PTPQ was in a usable and clear format, the final version consisted of five separate parts where each part starts as a new section and page, each written in bold letters:

A. General information and demographics

B. Practice Profile

C. Treatment Preferences

D. Bases for clinical work

E. Bases for educational/research work

At present the PTPQ is relevant to the Philippines and most likely in other developing countries (with minor changes as needed) in which physical therapy is not a first contact profession, or in a similar context of practice.

## Implications for research and practice

This study highlights the importance of taking the time to develop an appropriate instrument to match and collect the intended data for a research project. It outlines the processes related to the development and validation of an instrument which could be used by other researchers when developing similar questionnaires for other cultural groups. Table 3 is a summary of the practical applications of what the literature recommends in designing questionnaires (steps), the purpose and who are involved in each step, based on the actual methods done in this study.

**Table 3:** Practical applications in designing profile questionnaires

Steps	Purpose	Who are involved?
**Initial scoping**	• To check the availability of an appropriate instrument which matches the objectives of an intended study	• Authors
	• If no instrument is available, identify ways and possible sources of information on how to develop an instrument which will match the intended objectives of a study	• Researchers

**Focus group interviews**	• To explore key areas of concern in designing the instrument	• Key informants
		• Researchers

**Validation using the Delphi technique**	• To ensure that the instrument measures what it intends to measure	• Experts in different areas of practice
	• To collect highly generalisable data from the answers to be collected	

**Pilot testing**	• To "trial" the survey and identify possible problems to be encountered and allow troubleshooting to address the problems	• Participants representative of the sample population

The *Physical Therapy Practice Questionnaire (PTPQ) *was shown to have good face and content validity among the Filipino physical therapists and their context of practice. It is useful, easy to administer tool and in a format appealing to respondents. The PTPQ is expected to assist comprehensive data collection to create a profile of physical therapy practice in the Philippines or in other countries where a similar nature of practice is reflected.

## Consent Section

Informed written consents were received from participants in this study.

## Competing interests

The authors declare that they have no competing interests.

## Authors' contributions

JRD, KGS and SK contributed in the conceptualization, conduct and writing of this manuscript. All authors read and approved the final manuscript.

## Supplementary Material

Additional file 1 **PTPQ**. Full copy of the questionnaire. Click here for file
