# Supplementary material for: The physical therapy profile questionnaire (PTPQ): development, validation and pilot testing
Source: BMC Res Notes. 2011 Sep 19;4:362. doi: 10.1186/1756-0500-4-362 (PMC3182925; doi:10.1186/1756-0500-4-362)
Supplement: Additional file 1 — PTPQ. Full copy of the questionnaire. [file 1756-0500-4-362-S1.PDF]

The Physical Therapy Profile Questionnaire (PTPQ)

A. GENERAL INFORMATION SHEET

**Name (optional):**	**Age:**
**Current Work Position :**	**Gender:**<br>☐ **Female**<br>☐ **Male**
**Affiliation:**	
***Email address (for sending invites to trainings in the future)*:**	

Please fill in the following details about yourself:

Educational Background/Training

**Education**	**School/University**	**Location of School/ Region if within the Philippines; If outside the Philippines, please indicate which country**
Bachelors Degree		
Masters Degree<br>Please specify:		
PhD Degree<br>Please specify:		
Postgraduate (masters/PhD)units<br>Please specify:		
Certification/Diploma Course<br>Please specify:		

B. PRACTICE PROFILE

Please circle your answers to the following questions:

1. **How many years have you been in practice?**
   - A. < 2 years
   - B. 2- 5 years
   - C. 5 – 10 years
   - D. >10 years

2. **What is your current area of practice? (you can choose more than one answer)**
   - A. general practice
   - B. geriatric/neurologic
   - C. education
   - D. sports/musculoskeletal
   - E. pediatric therapy
   - F. cardiopulmonary rehabilitation
   - G. wellness/ health promotion
   - H. community development
   - I. research
   - J. others: ______________________________

3. **What is your current workplace setting/ environment? (you can choose more than one answer)**
   - A. hospital
   - B. private clinic
   - C. home care
   - D. community clinic
   - E. school/university
   - F. wellness/ sports facilities
   - G. multidisciplinary setting
   - H. others: ________________________________

4. **On a regular basis, how many professional colleagues do you work with in your workplace setting?**
   - A. 1- 5
   - B. 6-10
   - C. 11 – 15
   - D. >15

5. **The professional colleagues you work with in your workplace setting are: (you can choose more than one answer)**
   - A. PT practitioners
   - B. Medical doctors
   - C. OT practitioners
   - D. others: _______________________________

6. **Do you engage with colleagues to discuss patient cases or updates in practice?**
   - A. Yes
   - B. No

7. **If your answer to No. 6 is yes, how often do you have the discussions?**
   - A. weekly
   - B. monthly
   - C. as the need arises
   - D. others: _______________________________

8. **What are the roles you perform in your practice? (you can choose more than one answer)**
   - A. clinician/practitioner
   - B. administrator
   - C. educator/researcher
   - D. others: _______________________________

9. **On a weekly basis, how much time (in percent) do you spend for the roles you perform in Question 8? (e.g. 70% clinician, 30% administrator OR 50% administrator, 30% clinician and 20% educator)**

   ____

   ____

*** If you perform 100% administrative work, please stop here. Thank you for completing the survey**

**** If you perform ANY clinical work, please proceed to Sections C (Treatment preferences) and D (Basis for clinical work).**

*****If you perform ANY educational/research work, please proceed to Sections E (Basis for educational/ research work).**

Dizon, Grimmer-Somers and Kumar 2011

C. TREATMENT PREFERENCES

Which of the following treatment approaches do you often use and recommend in your practice? (you can choose more than one answer)

1. Manual Therapy techniques
_____ soft tissue mobilization techniques
_____ muscle energy techniques
_____ positional techniques
_____ Mulligan's technique
_____ massage
_____ others (specify): ____
____

2. Neuro developmental techniques
_____ Bobath exercises
_____ Brunnstrom technique
_____ Proprioceptive Neuromuscular Facilitation techniques
_____ Sensory integration techniques
_____ others (specify): ____
____

3. Cardiopulmonary therapy
_____ breathing exercise
_____ postural drainage
_____ ADL retraining
_____ lifestyle modification
_____ others (specify): ____
____

4. Therapeutic exercises
_____ stability exercises
_____ stretching exercises
_____ strengthening exercises
_____ endurance exercises
_____ others (specify): ____
____

5. Electrotherapeutic techniques
_____ Ultrasound
_____ Diathermy
_____ Electrical Stimulation
_____ TENS
_____ Infrared radiation
_____ Laser
_____ Hot packs /Cold packs
_____ others (specify): ____
____

6. Others (please specify):
______________________________ ______________________________
______________________________ ______________________________
______________________________ ______________________________
______________________________ ______________________________

D. BASIS FOR CLINICAL WORK

1. In your daily practice, what informs your decisions about treatment choices?
(Please rank from highest (1) to lowest)

_____ Undergraduate education
_____ Masters/PhD education
_____ Post graduate certification courses
_____ Seminars/ Conferences attended
_____ Hospital treatment protocol
_____ Recommendations from colleagues
_____ Doctor's prescription
_____ Journal articles/ research evidence
_____ Textbooks and other reference materials
_____ Experience
_____ Others (specify): ____

2. When you are faced with either a unique or new clinical case scenario, what informs your decisions about treatment choices?
(Please rank from highest (1) to lowest)

_____ Undergraduate education
_____ Masters/PhD education
_____ Post graduate certification courses
_____ Seminars/ Conferences attended
_____ Hospital treatment protocol
_____ Recommendations from colleagues
_____ Doctor's prescription
_____ Journal articles/ research evidence
_____ Textbooks and other reference materials
_____ Experience
_____ Others (specify): ____

3. Do you have access to the internet?

A. Yes
B. No

4. If you answered YES to Q3, how often do you use the internet to search for information about your clinical work?

A. always (76-100% of the time)
B. most of the time (50-75% of the time)
C. sometimes (less than 50% of the time)
D. never

5. Do you have access to the library?

A. Yes
B. No

6. If you answered YES to Q5, how often do you go to the library to search for information about your clinical work?

A. always (76-100% of the time)
B. most of the time (50-75% of the time)
C. sometimes (less than 50% of the time)
D. never

7. Are you familiar with databases and evidence based resources?

A. Yes
B. No

8. If you answered YES to Q7, which of the following databases do you search? (you can choose more than one answer)

A. Cochrane Library
B. Pub Med
C. Medline
D. PEDro
E. Science Direct
F. CINAHL
G. Google Scholar
H. others: ___________________________________

9. Which of the following do you look for when identifying the best approach for clinical cases? (you can choose more than one answer)

A. Clinical Guidelines
B. Systematic Reviews/ Meta Analyses
C. Randomized controlled trials
D. Any experimental study
E. Descriptive/ Observational studies
F. Others: __________________________________

10. Do you spend time to plan treatment for your patients?

A. Yes
B. No

11. If you answered YES to Q10, how much time do you spend in planning the treatment for each patient?

A. 1 – 10 minutes
B. 11– 30 minutes
C. 31- 45 minutes
D. 46 – 60 minutes

12. Do you conduct regular meetings/ case conferences in your workplace setting?

A. Yes
B. No
C. Not applicable

13. If you answered YES to Q12, who are the people involved in the meetings? (you can choose more than one answer)

A. PT practitioners
B. Medical doctors
C. OT practitioners
D. Others: __________________________________

14. If you answered YES to Q13, in what format do you conduct the meeting? (you can choose more than one answer)

A. case conference (SOAP of a case)
B. lecture presentations
C. journal presentation
D. others: __________________________________

15. What do you think are the trainings/ resources/ opportunities which will help you in your work?
(Please rank from highest (1) to lowest)

_____ Masters/PhD education
_____ Post graduate certification courses
_____ Seminars/ trainings
_____ Time to engage in the professional association
_____ Time and access to the literature/ databases/ research evidence
_____ Updated textbooks and other reference materials
_____ Exposure to local and international practice
_____ Others
(specify): ____

**** If you perform 100% clinical work, please stop here. Thank you very much for completing this survey***
****If you are a clinician with ANY educational or research work, please proceed to Section E (Basis for educational/research work)**

E. BASIS FOR EDUCATIONAL/ RESEARCH WORK

1. In your daily work, what informs your teaching content AND/OR research work?
(Please rank from highest (1) to lowest)

_____ Undergraduate education
_____ Masters/PhD education
_____ Post graduate certification courses
_____ Seminars/ Conferences attended
_____ Recommendations from colleagues
_____ Journal articles/ research evidence
_____ Textbooks and other reference materials
_____ Experience
_____ Others (specify): ____

2. When faced with either a teaching/ tutorial/ lecture/ research project which is unique or new to you, what is your best approach to search for information? *(Please rank from highest (1) to lowest)*

_____ Undergraduate education
_____ Masters/PhD education
_____ Post graduate certification courses
_____ Seminars/ Conferences attended
_____ Recommendations from colleagues
_____ Journal articles/ research evidence
_____ Textbooks and other reference materials
_____ Experience
_____ Others (specify): ____

3. Do you have access to the internet?

A. Yes
B. No

4. If you answered YES to Q3, how often do you use the internet to search for information about your educational/ research work?

A. always (76-100% of the time)
B. most of the time (50-75% of the time)
C. sometimes (less than 50% of the time)
D. never

5. Do you have access to the library?

A. Yes
B. No

6. If you answered YES to Q5, how often do you go to the library to search for information about your educational/ research work?

A. always (76-100% of the time)
B. most of the time (50-75% of the time)
C. sometimes (less than 50% of the time)
D. never

7. Are you familiar with databases and evidence based resources?

A. Yes
B. No

8. If you answered YES to Q7, which of the following databases do you search? (you can choose more than one answer)

A. Cochrane Library
B. Pub Med
C. Medline
E. Science Direct
F. CINAHL
G. Google Scholar

D. PEDro
H. others: ___________________________________

9. Which of the following do you look for when identifying the best approach for clinical cases? (you can choose more than one answer)

A. Clinical Guidelines
B. Systematic Reviews/ Meta Analyses
C. Randomized controlled trials
D. Any experimental study
E. Descriptive/ Observational studies
F. Others: ____________________________________

10. Do you spend time to plan your educational/ research work?

A. Yes
B. No

11. If you answered YES to Q10, how much time do you spend in planning your work?

A. < 30minutes
B. 1 – 3 hours
C. half day
D. one whole day
E. others: ____________________________________

12. Do you conduct regular meetings/ planning sessions in your workplace setting?

A. Yes
B. No
C. Not applicable

13. If you answered YES to Q12, who are the people involved in the meetings? (you can choose more than one answer)

A. PT practitioners
B. Medical doctors
C. OT practitioners
D. Instructors/ professors
E. Others: ___________________________________

14. If you answered YES to Q13, in what format do you conduct the meeting? (you can choose more than one answer)

A. case presentation
B. group discussion
C. lecture presentation
D. others: ___________________________________

15. What do you think are the trainings/ resources/ opportunities which will help you in your work? ***(Please rank form highest (1) to lowest)***

_____ Masters/PhD education
_____ Post graduate certification courses
_____ Seminars/ trainings
_____ Time to engage in the professional association
_____ Time and access to the literature/ databases/ research evidence
_____ Updated textbooks and other reference materials
_____ Exposure to local and international practice
_____ Others
(specify): ____

**** Thank you very much for completing this survey.***
